# Intimate partner violence linked to gambling: cohort and period effects on the past experiences of older women

**DOI:** 10.1186/s12905-023-02316-0

**Published:** 2023-04-06

**Authors:** Nerilee Hing, Catherine O’Mullan, Lydia Mainey, Elaine Nuske, Helen Breen

**Affiliations:** 1 Central Queensland University, G.07 Building 8, University Drive, Bundaberg, QLD 4670 Australia; 2 Central Queensland University, 42-52 Abbott Street & Shields Street, Cairns City, QLD 4870 Australia; 3 Southern Cross University, Gold Coast Airport, Terminal Dr, Bilinga, QLD 4225 Australia; 4 Southern Cross University, Military Rd, East Lismore, NSW 2480 Australia

**Keywords:** Intimate Partner violence, Problem Gambling, Older women, Cohort Effects, Period Effects, Service Provision

## Abstract

**Background:**

Problem gambling increases the risk of experiencing intimate partner violence (IPV). People impacted by gambling-related IPV face distinctive challenges, and these may be compounded by intersections with gender, generational influences and contextual factors. This study explored the past experiences of older women affected by male partner violence linked to gambling, and how these were shaped by cohort and period effects and problem gambling. Cohort effects are the generational characteristics of a group born at a particular time, while period effects relate to prevailing external conditions at the time of the abuse, including laws, services and practices.

**Methods:**

A larger study exploring the nature of the relationship between problem gambling and IPV recruited 72 women through help services and advertising. The current study analysed a subset of interviews with 22 women aged 50 years or over. We analysed the data using adaptive grounded theory to explore the intersection between IPV, gambling, and cohort and period effects.

**Results:**

Cohort effects on the women’s experiences of IPV included gendered attitudes, traditional views of marriage, silence surrounding IPV, reticence to disclose the abuse, and little understanding of problem gambling. These influences deterred women from questioning their partner’s gambling, and to instead keep the gambling and abuse hidden. Many women did not recognise abuse linked to gambling as IPV, since gambling was considered a normal, harmless pastime. Having a gambling problem exacerbated violence and coercive control by male partners as traditional gender norms supported male authority over their female partner. Women with a gambling problem sometimes felt they deserved the abuse. Period effects included a lack of IPV and gambling services, gendered service responses, failure to prioritise the women’s safety, and no consideration by services of the role of gambling in the abuse.

**Conclusion:**

Reducing gender inequality is critical to reduce male partner violence towards women. Women impacted by gambling-related IPV, including the legacy of past abuse, need service responses that recognise all forms of abuse, understand the historical and contextual factors that exacerbate it, and recognise how gambling can amplify IPV. A reduction in problem gambling is needed to reduce gambling-related IPV.

## Background

Intimate partner violence (IPV) encompasses behaviour by an intimate partner or former partner that causes physical, sexual or psychological harm, including physical aggression, sexual coercion, psychological abuse and controlling behaviours [1]. Several factors intersect with experiences of IPV, including race, class, age, sex and gender [2]. In addition, reinforcing factors, such as the harmful use of substances or gambling, can increase the frequency and severity of IPV perpetration [3]. This study focuses on how the intersection between IPV, problem gambling, and older women’s earlier experiences impacts on their lived experience of abuse. Understanding the interplay between these factors can help to inform research, policy and practice. The paper first reviews links between gambling problems and IPV, and then how cohort and period effects shape women’s experiences of IPV.

### Gambling problems and IPV

Research demonstrates a consistent, significant relationship between problem gambling and IPV. In a review of 14 studies, 36.5% of people experiencing problem gambling acknowledged perpetrating physical IPV, while 38.1% had been subjected to physical IPV [4]. In a representative US survey (*N* = 25,631), having a gambling problem nearly tripled the likelihood of physical IPV [5]. These rates would be higher if all types of IPV were measured, including psychological, sexual, verbal and financial abuse and patterns of coercive control [6].

Several factors heighten the risk of IPV perpetration among people experiencing problem gambling [6]. These include the stress that gambling creates, which leads to financial, emotional and relationship pressures that increase the risk of violent incidents, particularly when a person’s gambling losses fuel their anger and frustration [7–10]. Mental health and substance use disorders, which have elevated co-occurrence with problem gambling, further exacerbate the risk of IPV perpetration [7, 11, 12]. Gambling can also interact with gendered drivers of violence, such as patriarchal expectations of hierarchical power and control in a relationship [13–16]. This can extend the abuse to include control over the partner’s finances, coercion to provide gambling funds, and use of violence to vent anger, frustration and blame about gambling outcomes [17–19]. Behavioural drivers associated with gambling addiction also exacerbate IPV. Reflecting the preoccupation with gambling, strong gambling urges, erroneous cognitions and withdrawal symptoms that characterise problem gambling, gamblers can direct their exasperation at their partner when they lose or are unable to gamble [19, 20].

Several factors elevate the risk of IPV perpetration against people with a gambling problem [6]. Intimate partners typically report shock, betrayal, anger and fear when a gambling problem is revealed [21–24]. Financial stress is often already acute and may reveal substantial debt, loss of lifetime savings, or the need to sell the family home [20, 22]. This financial devastation and realising their partner’s prolonged deceit typically cause significant distress. Ongoing cycles of continued gambling, quit attempts and relapse can cause partners prolonged stress, with accumulating anger, mistrust and conflict increasing the risk of violence [10, 25].

Gambling can also be a response to violence. Some women start or escalate their gambling to cope with severe and chronic IPV – to physically escape by going to gambling venues, to psychologically escape from the IPV trauma, or to cope with the legacy of past abuse after separating from a violent partner [26]. Women seeking this solace most often gamble on electronic gaming machines, which pose substantial risk of dependency [26–29]. This gambling can be weaponised to excuse further violence and control, leading to a self-reinforcing cycle where the gambling and violence escalate [26].

### Older women’s past experiences of IPV: Cohort, period and gendered effects

Little is known about the intersection between gambling and IPV experienced by women from earlier generations. Nonetheless, cohort and period effects that stem from the social historical period in which they grew up are likely to have shaped their experiences [30].

Cohort effects are the generational characteristics of a group born at a particular time and can influence the experiences of IPV amongst women [30]. Research in 25 low and middle income countries found that the oldest cohort of women (born between 1958 and 1967) was less likely to report their experiences of IPV and more likely to consider IPV to be justified in certain circumstances [31]. As a cohort, these older women’s experiences reflect the socio-political context that helped foster their first ideas about gender norms and attitudes about IPV [31]. This includes their socialisation into traditional values that encourage women to be submissive to their husband, silently accept their situation, maintain family solidarity, and avoid the taboo of divorce [30–32]. The expectation that marriage was forever, and the prevailing social norms, meant a woman’s identity was strongly tied to her marriage and she had high levels of psychological, social and financial dependence on her husband [30, 33]. Leaving an abusive relationship therefore incurred high costs for women, including shame, loss of social networks, reduced social status, and financial stress if they did not have an independent income [30, 34]. In addition to social stigma, divorce was likely to bring loneliness, little prospect of re-partnering, few recreational opportunities and limited social networks [32, 34]. Abuse often leaves women with low self-esteem and feelings of self-blame, hopelessness and powerlessness, further reducing their capacity to leave or seek help [35].

Period effects refer to history, events and prevailing external conditions at the time of the abuse, including laws, services, policies and practices [30]. Women in earlier generations were deterred from seeking help for IPV because they had low expectations that other people or services would be helpful [33, 34]. Beaulaurier et al. [35, 36] describe how disclosures of abuse to family and friends often prompted victim-blaming, denial of the abuse, or opposition to breaking up the family. External barriers to help-seeking included expectations that justice system responses would be biased towards the abuser and worsen the situation, and a lack of support services. Responses from other people and services are a critical influence on women’s future help-seeking behaviours, but older women typically report they were negative and unhelpful at the time they sought help [32, 34, 37].

Central to the cohort and period effects on women’s experiences of IPV is the gendered nature of power and control in relationships, gendered service responses, and societal mores that uphold gender inequality [30–34, 38]. Gender inequality has been pronounced throughout the lives of today’s older women, permeating their self-identity and relationships, the services and systems they have interacted with, as well as social norms [8]. Gender inequality underpins IPV against women [33, 39, 40], so this study was sensitive to considering its role in their experiences of gambling-related abuse.

### Older women’s past experiences of gambling-related IPV

Little is known about the earlier experiences of gambling-related IPV among older generations of women. How these issues might intersect appears to be complex. As discussed earlier, there are several mechanisms by which gambling problems can exacerbate IPV and IPV can exacerbate gambling. These are likely to intersect with the cohort and period effects experienced by older women during their lifetime, including gender norms pertaining to intimate relationships, service responses and societal expectations.

To help to address this gap in understanding, this study aimed to explore the past experiences of older women who have experienced male partner violence linked to gambling, and how these were shaped by cohort and period effects at the time of their abuse and by the nature and impacts of problem gambling.

## Methods

### Recruitment and participants

This study analyses a subset of interviews conducted for a larger study into the nature of the relationship between gambling and IPV against women, including gambling by the male or female partner [18]. This larger study interviewed 72 women subjected to gambling-related IPV, five men who had perpetrated gambling-related IPV, and 39 service providers with relevant experience in their DFV, gambling, financial counselling, and culturally-specific services. After ethical approval from the lead university’s Human Research Ethics Committee (approval number: 20,852), we asked the 39 service providers to advertise the study to suitable clients, either by personal communication or making recruitment flyers available in their service. We also advertised on Google, Gumtree and Gambling Help Online, and through emails to previous research participants who had consented to receive information about future studies. Potential participants could register their interest on a project-specific website or by contacting the project officer via telephone, email or SMS. The project officer contacted each potential participant to check their eligibility and organise an interview time. Inclusion criteria were: aged 18 years or over; had engaged with a support service for IPV and/or gambling; and had lived experience of IPV from a male partner related to gambling. IPV was defined to participants as “acts of violence that occur between two people who are, or were, in an intimate partner relationship. It includes physical, sexual, emotional, psychological and financial or economic abuse” [41]. Of the 72 women interviewed, 52 identified how they found out about the study: referrals from help services (37%), Gumtree advertisements (27%), Google Ads (23%), emails to previous research participants (8%), advertisements on Gambling Help Online (4%), and a friend (2%). The non-probability sample may not be representative of the population of women affected by gambling-related IPV. Detailed materials and methods for the larger study are available [18].

Of the 72 women in the larger study, 22 were aged 50 years or over and are the focus of this analysis. Specifically, 13 were aged 50–59 years and nine were aged 60–69 years. Thirteen of these women had experienced IPV in the context of their male partner’s gambling problem (WMG: woman, man’s gambling), and six of these 13 women chose to focus their interview mainly on his financial abuse (WFA: woman, financial abuse). An additional nine women experienced IPV in the context of their own gambling problem (WWG: woman, woman’s gambling). All women had experienced IPV when younger and all except five had terminated the relationship.

### Data collection

After gaining informed consent, three authors conducted telephone interviews with participants, lasting 30–120 min. To allow participants to speak freely, the interviews were unstructured, simply asking them to recount how gambling and abuse from a partner had affected their life. While the interviewers could draw on predetermined prompts to help ensure relevant issues were covered (e.g., the nature of the abuse, the gambling and its impacts, help-seeking behaviour), the participants generally provided detailed accounts with little prompting. Participants were compensated with a $40 shopping voucher.

Several strategies were used to minimise the risks of causing distress to participants and exacerbating the IPV. The participant information sheet explained potential risks and listed contact details of DV, gambling, financial counselling and general support services. To ensure participants had access to professional support if needed, the team only recruited participants who had previously sought professional support for IPV or gambling. During recruitment, participants were offered several options for contacting the research team, as outlined above, and could opt to use a pseudonym in all communications. The research topic was purposely kept vague during direct, pre-interview contact to protect participants’ privacy and safety, particularly from perpetrators who may overhear or read communication. Participants were asked to ensure they were in a private space during the interview where they could not be overheard or interrupted, particularly by perpetrators. They could opt for a support person of their choosing to accompany them, but not the perpetrator.

The interviewers (CO, LM, EN) had extensive experience conducting sensitive interviews in their prior practice and research on DFV, sexual assault, problem gambling and social work. In preparation for the project, they undertook additional training on DFV, conducting sensitive interviews, intersectionality and gendered issues in DFV. They took numerous steps to avoid retraumatising participants. These included building rapport, careful listening, allowing participants to direct the interview, avoiding rushing or interrupting participants, and “backing off” if topics became particularly painful. If participants experienced distress, interviewers responded empathically, validated their experiences, and moved onto more positive topics if necessary. Participants were offered repeated opportunities to take a break, stop the interview, or withdraw from the study. Some interviews were conducted over two or three sessions if the participant wanted to share more. At the end of each interview, the interviewer conducted an informal debriefing, provided contacts for support services, and invited participants to contact them if they wanted to talk further.

### Analysis

The analysis used an adaptive grounded theory approach [42] that combined inductive processes of the grounded theory method [43] with deductive analysis informed by existing theory. After a professional service transcribed the interviews, the lead author used open coding to systemically code relevant portions of the transcripts to create initial codes of potential relevance to the research aims. She then used a further iterative process to identify emergent themes, using the constant comparative method to continuously add new codes, modify existing ones, and recode data as needed. Next, all authors reviewed the coded data to identify similarities and overlaps, and collaborated to group codes with common features to create meaningful themes and sub-themes in the data. Through an iterative process of review and revision, the authors finalised the themes and sub-themes. Finally, two authors (NH, HB) reviewed the interview transcripts and included participant quotes to further enrich and support the themes.

In line with the adaptive grounded theory method [42], the analysis also involved deductive processes. In recognition that existing knowledge aids interpretation [42], our analysis was sensitive to the widespread recognition that gender inequality provides the fundamental context within which men perpetrate IPV against women [1, 31, 40]; and that experiences of IPV are influenced by cohort and period effects, as reviewed earlier. Because influences on IPV occur at multiple levels of the social ecology [3, 37], we differentiated between individual and relationship, systemic and societal influences in our analysis. The analytical approach therefore engaged with both emergent patterns in the data and existing concepts and theories.

## Results

Participants reported a wide range of abuse from their male partner, including emotional and verbal abuse, stalking and threats, physical assaults including some that required hospitalisation, and sexual violence. Where their male partner had a gambling problem, he also subjected them to financial abuse to pay for his gambling and gambling debts. The women who had a gambling problem usually started frequenting gambling venues to physically escape their partner’s abuse and emotionally escape by playing electronic gaming machines. The male partner often used her gambling to justify his violence, which typically escalated with her gambling losses. Most women reported prolonged periods and intensifying cycles of violence, including cumulative patterns of coercive control.

Table 1 summarises the key influences we identified on the women’s experiences of this violence, presented by cohort and period effects, in three socio-ecological levels, and whether they related most to generational/contextual effects or gambling effects. These are discussed below and supported by interview quotes.

**Table 1:** Major influences and effects on the older women’s past experiences of gambling-related IPV

Cohort or period effect	Socio-ecological level	Influences on older women’s experiences of gambling-related IPV	Effects of generational and contextual factors on IPV	Effects of gambling on IPV
Cohort effects	Individual and relationship influences	Gendered attitudes and behaviours amongst the women	Acceptance of traditional gender norms within the relationship led to self-blame, self-sacrifice, and acceptance of the situation, including the abuse.	Acceptance of traditional gender norms within the relationship also meant not questioning his gambling and the associated abuse.
		Gendered attitudes and behaviours amongst male partners	Acceptance of traditional gender norms within the relationship led to a perceived entitlement to control decision-making, subordinate their partner, and use violence against her.	The gambling addiction exacerbated the individualistic and controlling behaviours of male partners and provided a strong motivation for coercive control and financial abuse.
		Reticence to disclose the abuse to family and friends	Expectations to keep domestic problems private meant that most women kept the abuse hidden due to shame, and an expectation that family and friends would not be helpful.	Expectations to keep domestic problems private meant that most women kept the gambling problem hidden due to shame, and an expectation that family and friends would not be helpful.
Cohort effects	Societal influences	Traditional gendered views of marriage	Women’s socialisation into traditional gendered norms in marriage pressured them to maintain the façade of a perfect family by tolerating and concealing the abuse.	Tolerating and concealing domestic problems also included gambling problems.
		The silence surrounding IPV	Lack of public discourse about IPV meant women often did not recognise the behaviour as abuse or saw it as a normal part of relationships.	Lack of public discourse about IPV meant women did not recognise gambling-related abuse as IPV; and women with a gambling problem could feel they deserved the abuse.
		Little societal recognition of problem gambling	No public discourse about problem gambling.	Gambling was seen as a normal and harmless pastime by the women, family and friends, and institutions such as the police and justice systems.
Period effects	Systemic influences	Lack of IPV and gambling services	No IPV services, financial support or childcare existed to help women escape violent relationships.	No gambling help services existed for gamblers or their partners to help address the gambling problem.
		Unhelpful, enabling and gendered service responses	Little help for women experiencing IPV as service responses included victim-blaming, stereotyping women as hysterical, and a failure to take the abuse seriously.	No consideration by services of the role of gambling in the abuse.
		Failure to help protect the woman’s safety	Limited understanding of IPV by services could result in a failure to protect the woman’s ongoing safety.	No consideration by services of the role of gambling in the abuse.

### Cohort effects at the individual and relationship level

#### Gendered attitudes and behaviours amongst the women

Many women initially blamed themselves for problems in the relationship and tried harder to make it work, downplaying their own needs to meet their partner’s demands. Some women assumed personal responsibility for the abuse: “I must be doing something wrong. That’s why this is happening” (WMG007). They often felt that even more sacrifice was needed to be considered “a good enough woman” (WMG078).

At the time of their interview, five women remained in their relationship after years of abuse. One woman’s partner had resolved his gambling problem so his financial abuse had eased. The abuse had not diminished for the other four women. They reported a continuing lack of connectedness and seemed resigned to living separate, emotionally detached lives under the same roof:

We’re basically cohabiting in the same house now, and he has certain duties, and I have certain duties, and we kind of cross in the night. I’m not sure what sort of relationship you’d call this. I call it life. (WWG019)

Women also stayed to hold onto the couple’s shared history and financial security: “you work all your life to get some security, and we have that now. We’ve got a home which has no mortgage” (WWG019). Women also spoke about staying married for the sake of the children and to maintain the façade of a happy home:

It doesn’t matter what we’re going through as long as the kids are happy, as long as mum and dad and grandma and pop and everyone else thinks it’s a rosy world, just go along with it. (WWG038)

Women who had eventually left their abusive partner also related how they had stayed a long time for their children’s sake, because they “deserved [to have] their father” (WWG024), and “so I could keep my children safe” (WMG001). Some women feared they might lose custody of the children if they separated, or that the father would not provide appropriate care when they stayed with him under the equal shared responsibility arrangements favoured by Australian courts. Women described a range of reasons for ending the relationship, including anger, exhaustion, fear of being killed, and a “last straw” event including his infidelity, a particularly violent incident, or him gambling all the family’s remaining money.

Women are more likely to experience IPV if they hold attitudes accepting of male privilege and women’s subordinate status [1]. These perspectives were evident in the women’s self-blame and self-sacrifice in the face of their partner’s violence. The shared history and security provided by marriage meant that some women accepted their situation. The welfare of children was a powerful reason for the women to stay or stay longer.

#### Gendered attitudes and behaviours amongst male partners

The women typically described their partners as misogynistic, controlling, entitled and selfish. They described their hierarchical relationship where he “always put himself first” (WMG078) and being treated with contempt: “sarcastic…so obnoxious…just belittling me” (WFA021). This humiliating act reflected one partner’s extreme disrespect: “He pinned me down on the floor with one arm, and…pulled out his penis and urinated on me at the same time” (WWG017). A comment from one husband encapsulated his misogynistic attitude which he explained through the lens of cultural norms: “Indian women should stick to their husbands, should be standing next to their husbands helping. Not blaming or leaving” (WMG007).

The women invariably described their partner as controlling, setting rules and boundaries: “I wasn’t allowed to answer the phone” (WWG017), “he’d take my [car] key…he didn’t want me to leave…Because he has the power then” (WWG024), and “if I wanted to go and see a friend…I had to seek approval” (WWG012). Male partners were often described as demanding, “a perfectionist” (WWG067), who expected the woman to cater to his needs and felt entitled to abuse her if something did not please him.

Having a gambling problem appeared to amplify these controlling and abusive behaviours, especially in relation to the family’s finances: “he always had control of the money…he embezzled the relationship…if I was his boss he’d be in gaol” (WFA003). Another woman commented:

Any money that he wasn’t controlling or he wasn’t spending, or he wasn’t wasting on gambling…he was always angry and resentful about money outside of what he could control or spend. (WFA006)

The symptoms of a gambling addiction exacerbated the individualistic tendencies of these men, as they became preoccupied with and irrational about their gambling, and experienced strong urges to gamble, tension if unable to gamble, and frustration at their continued losses. One woman described: “I’d come home [from work], the children would still be in their pajamas and starving at 6 o’clock at night, because he would have been absorbed in the gambling” (WFA006). Women were often blamed for their partner’s gambling losses:

He really was obsessed with thinking he knew the final outcome of the race, and if I didn’t hand it [money] over, I was somehow being abusive to him and our family, and I was stopping us from having a win. (WFA027)

These characteristics of abusive partners align with those typical of IPV perpetrators [26]), and reflect common gendered drivers of violence against women. These include where men feel entitled to use violence against women, have rigid gender expectations and roles, maintain control over decision-making, and consider women to be subordinate to men [3]. Having a gambling addiction intensified the abuse as the obsession with gambling, irrational beliefs, and need for money overrode the family’s welfare and fuelled tensions and conflict.

#### Reticence to disclose the abuse to family and friends

The women faced substantial barriers to disclosing their abuse which most often “stayed behind closed doors” (WFA027) at that time. One woman noted that her partner’s friends would not challenge him about his abuse because “they were doing the same thing to their missus” (WMG002). She described the shared silence with other wives whom she suspected were also experiencing IPV:

We would kind of have a solidarity between all the wives that would be sitting outside the pub waiting for their husbands to come out, and, you know, hopefully have some skerrick of their pay left so that we could pay bills. (WMG002)

Reflecting the social isolation that is common amongst abused women, some women referred to avoidance, becoming “insular” (WMG001) and “I cut myself off completely” (WWG017). Some who did disclose the abuse were not believed, which further undermined their confidence:

They just didn’t think he had it in him because he was such a placid person…it’s not really good when your closest friends don’t believe it…you sort of start thinking “is it my fault?”. (WWG045)

Family and friends also had less knowledge of problem gambling a few decades ago, with gambling seen as a relatively harmless pastime that was not linked to abuse: “no one knew…the impact of gambling…that you could get addicted” (WMG007). Another participant explained:

They all thought he was wonderful. But a lot of them knew he was gambling; more than I knew. But…Everybody gambles. Everybody puts money through the pokies. But they didn’t understand the gravity of it. (WFA015)

While some women reported supportive responses from family and friends, most kept the abuse and gambling problems hidden due to shame, and an expectation that family and friends would not believe them, trivialise the abuse, blame the woman, or pressure her to remain silent.

### Cohort effects at the societal level

#### Traditional gendered views of marriage

In line with social norms at the time, the participants often expressed traditional views about the permanence of marriage and how children should be brought up only within marriage. These values increased the pressure they felt to stay in the abusive relationship because “this is my lot” (WWG064) and “Death us do part, that’s it” (WWG019). Some interviewees clearly felt family and social pressures to marry and to stay married, especially if they were older or had previously been divorced:

I was getting older…and I thought, “Right, I’m going to marry this dude.” And in the back of my mind is, “Yeah Mum, I can get married. Someone does love me.” (WMG005)

Many women reflected on their upbringing when societal norms conveyed rigid gender roles within marriage, and wives fulfilled subordinate homemaker roles and catered to their husband’s needs. One interviewee explained: “I’ve been kind of raised with very Victorian attitudes that…lend themselves to me being kind of a bit submissive…and just too trusting”, which made her “an easy target” for the abuser (WFA003). Parents often conveyed that women should cater to their partners’ needs even if it constituted abuse, and to suffer in silence: “If anything happens, keep it to yourself and try to fix it” (WMG007). One woman experiencing sexual abuse from her husband told her mother:

“I’m telling you it’s like enforced prostitution. I can’t get over it.” You know what she said to me? She put her hand on my arm. She looked absolutely so sad and she said, “Darling, women did it during the war. Don’t worry.” So I came from a long line of coping, suffering sorts of women. (WWG003)

This submissiveness and silence also extended to a partner’s gambling problem which was not to be discussed or complained about, and instead just “do whatever you can to keep him happy” (WMG001):

In my generation your mother said, “You make your bed, you lay in it, don’t come home with your problems. If you get married, that’s it, that’s your problem”…So you didn’t kind of talk about it. (WMG002)

One woman compared the generational tendency for women in her age cohort to be submissive, to changing attitudes amongst younger women:

Hopefully women have got a bit more backbone than what we’ve had because we were subservient…because women are realising that they do have equal powers. For people of our age group, we’re sort of stuck in the time zone. (WWG038)

The women’s perspectives reflect their socialisation during upbringing into attitudes and values based on traditional gendered norms in marriage, and the importance of maintaining the façade of a perfect family by tolerating and concealing domestic problems, including IPV and gambling.

#### Silence surrounding intimate partner violence

Socialisation into traditional gender roles and the shame of disclosing marital problems can prevent women from recognising and reporting IPV [31, 44]. IPV legislation was not introduced and rape within marriage was legal until the 1980s. Thus, the participants often did not recognise at the time that their partner’s behaviour constituted abuse. For some women, this realisation did not occur for many years, until IPV attracted increased publicity and public censure [45]. As one woman expressed, “I never realised that what was happening to me was extreme domestic violence…I’ve only really, like, in the last couple of years decided about how bad the DV was” (WMG002).

Other women had thought IPV constituted only physical abuse, where “you got a black eye” (WWG038). Many women did not realise that emotional, verbal and financial abuse also constituted IPV. This led to accepting the behaviour or, downplaying its effects:

I just thought I was in a poor relationship, where there wasn’t much money. I didn’t really understand that I was being deprived of the basic needs of life, because I was in a financially abusive situation. (WFA027)

Some women explained that their partner’s diagnosis of other health issues, including problem gambling, obscured their recognition that his behaviour constituted IPV, leading to more sympathetic attitudes:

It took six weeks to convince me that it was domestic violence, because I had listed it as, “DSM-3 addiction to gambling”…And you go, “Well, I can’t treat them any differently to I would as a cancer patient, because mental health is an illness”, and therefore then you’re stuck. (WMG084)

Women suffering physical abuse did not always see this as IPV or accepted it as a normal part of relationships or cultural norms: “Unfortunately it’s part of culture where Aboriginal women think if they get flogged up, well, then their man loves them and wants them” (WMG002). Women with a gambling problem might feel they deserved the abuse, particularly if their partner used their gambling to justify his violence:

I was still blaming myself all the way, and then it was only at the very end, [when I saw a roadside sign saying] “If he bashes you leave”…I had my first realisation of violence. (WWG003)

One interviewee contrasted the greater willingness to talk about IPV now compared to the prevailing silence during her earlier life: “whereas, once upon a time you just wouldn’t bring it up” (WFA027).

The silence around IPV at the time meant these women often did not recognise their partner’s behaviour as abuse, especially if it did not involve physical assault. Financial abuse to fund his gambling was also not recognised as IPV. They might also excuse his violence if he had mental health issues or downplay it as a normal part of relationships.

#### Little societal recognition of problem gambling

At the time of the women’s abuse, there was little societal recognition that gambling could become problematic. Gambling “was just something that people did” (WMG001), a recreational pastime that people were not familiar with and did not realise could fuel abuse:

I didn’t understand the gravity of a gambling problem…It’s access to money, and it doesn’t matter whose money it is…Not knowing it’s a lifelong illness. It’s not something that gets fixed. (WFA015)

The women found that the police and justice systems had little understanding of problem gambling, or the financial abuse it often leads to. One woman said “I was taken aback and horrified at how the Family Law system dealt with gambling and treated it as a hobby” when she presented records indicating her partner spent around seven hours each day on online gambling and had spent $100,000 (WFA006). Another woman related:

The policemen…just wrote down, “oh there’s gambling losses” or whatever. And I realised that the police had no idea about what they were dealing with, because when you go to the court, they have signs up saying that there’s help for alcohol and drug treatment. But they had no gambling treatment in the court system. (WFA012)

One woman compared the greater publicity about problem gambling in contemporary Australia to when she was young, which has helped people recognise its serious consequences:

I’ve seen the ads on TV and stuff about responsible gambling. That’s probably a really big step because it is seen as a problem. Whereas, back in the day it really wasn’t seen as a problem, it was seen as a hobby. (WF027)

Problem gambling as a public health issue did not enter the public discourse until about 20 years ago. People therefore had little knowledge of its symptoms, impacts and potential to exacerbate IPV.

### Period effects at the systems level

#### Lack of IPV and gambling services

Family violence services did not exist at the time the women were experiencing IPV: “Honestly, there wasn’t anything” (WMG002). Lack of safe refuges and affordable accommodation were strong deterrents to leaving. All women with a partner who gambled faced severe financial distress, which further hindered their escape since no financial support was available: “no [child] endowment…no Centrelink…if you didn’t work you got no money” (WMG002). With no childcare, working was difficult for women with young children, and could deter them from leaving or result in them returning to their abusive partner:

I had three little kids, trying to work full time shift work with no family to help me, and there was no childcare…So consequently I took him back and he kind of developed the gambling habit then. (WMG008)

Gambling help services were also non-existent: “GA [Gamblers’ Anonymous] was very unheard of…But in [small town], there was nothing in phone books or anything” (WFA015); and “I don’t even recall there being a helpline back in my day” (WFA027).

This lack of services reflects the prevailing conditions when these women’s IPV experiences were escalating, with few support systems for women seeking to escape violent relationships or to address gambling problems in the family.

#### Unhelpful, enabling and gendered service responses

The women’s narratives frequently exposed the subordination of women’s needs, gendered responses, and a limited understanding of IPV at that time by some services. Healthcare professionals sometimes blamed the woman. One woman who presented to her male doctor with a battered face from her partner was told “Well, you probably deserved it” (WWG003). Other women had unhelpful experiences with counsellors who left them feeling misunderstood, powerless, stigmatised and with no plan of action, perhaps reflecting less understanding at the time of strengths-based approaches to therapy [46, 47]:

I don’t remember this person helping at all. She didn’t seem to understand anything…I want to say she was completely useless…I walked out of there feeling no better, like no more decisive, no more valued, nothing…I got no help. (WMG001)

Most women who had turned to the police reported negative and hurtful responses. One woman recalled that the police failed to act even though her partner had made death threats against her:

They [the police] could have taken the DVO order out on him…[but] it was a real effort on their behalf, you know, that I was just being a nuisance and…it’s not unless he’s actually punching you in the face or breaking your bones or kicking you down the stairs or something, then you don’t really have anything to whinge about…even though he told my 15 year old that…he was arranging to have somebody kill me. (WMG008)

Other women recalled that the police treated them as hysterical women who were an annoyance. The police treated one woman as the main perpetrator when she accidently scratched her partner’s face when she tried to deflect his hold on her:

The police treated me like absolute dirt. They removed me from the house and took me to the police station, did an interview, took my fingerprints…I was crying and I said, “I don’t know where to go”. He said, “if you keep crying, I’ll drop you off at the mental hospital”. (WWG024)

In some cases, the police deterred the women from laying charges against the perpetrator, warning her it would bring shame to the family. In the example below, this woman had video evidence that her partner repeatedly drugged and raped her. She asked two doctors for a test to confirm he was drugging her; neither doctor recorded her requests in her medical records. Lacking this evidence, the police discouraged her from pressing charges:

I tried to press for sexual assault claims, but the police advised me not to…because I was drugged, and they couldn’t tell whether I was participating or not participating. (WMG084)

The women reported overwhelmingly negative experiences when they sought help from most services, including victim-blaming, stereotyping them as irrational, and failing to take their abuse seriously. Gender inequality, upheld by mainly male doctors, police officers and justice personnel, and lack of professional understanding of IPV, shaped these responses.

#### Failure to help protect the woman’s safety

The women related several instances where services failed to assist in protecting their safety. One interviewee and her husband went to the doctor specifically to discuss his abusive behaviour. The doctor took no follow-up action to try to protect the woman or steer the husband towards behavioural change:

I remember the GP saying to him, “can you hear what your wife is saying?…She’s really upset, and she’s really affected by your actions…have you got anything to say about how you’re making her feel?” He just said, “I’m sorry”. Sorry, that was it…the practice could have phoned [partner] directly…for a follow-up appointment, or phoned me to say the same…Any of those would have been helpful, but to hear nothing… (WWG067)

Some women noted that healthcare professionals were unlikely to ask if IPV was occurring or if the woman was safe, and none asked about gambling. This comment reflects a disconnect between medical and psychological services at the time: “When you go to the doctor and you’re crying all the time…Rather than slip somebody an antidepressant pill, try and find out what the problem is and offer some counselling” (WFA027). Another woman commented that ambulance drivers in her small town missed the opportunity to check on people’s safety:

The ambos…working in the community for years and they know you get banged around. But even they wouldn’t say, you know, “are you safe or…do you need somewhere to go?” (WMG002)

Several women commented on the failure of the court system to prioritise their safety and the weak deterrence of domestic violence orders (DVOs): “the judge gave him an intervention order for 12 months and he was back on my doorstep that night” (WWG024). One woman related how a judge refused to renew a DVO, determining that: “we’d been separated long enough and that we needed to learn how to manage our conflict” (WMG008). Even when women and their children were facing an immediate threat, services might ignore obvious risks to their safety:

I ended up jumping out of the car to get away from him. Then…he went home and…took off with [our young child] out in the bush and I called the police…they said, “Oh, look, he won’t do anything to him. He’s his father.” (WMG008)

These women’s experiences reflect the limited understanding of IPV amongst professionals at the time their abuse was escalating, resulting in a failure to protect their safety. Even though services may have improved, the women’s negative experiences most likely continued to shape their attitudes to help-seeking.

## Discussion

This study has explored the earlier experiences of older women who have been subjected to male partner violence linked to gambling, and how these were shaped by cohort and period effects and problem gambling. Key findings demonstrating the intersection between IPV, gambling problems, and these older women’s past experiences (Table 1) are discussed below.

Cohort effects at the individual and relationship level were characterised by the gender inequality that permeated the experiences of this cohort of women, highlighting its role as a fundamental contributor to IPV [33, 39, 40]. The women’s marriages assumed men’s elevated status and authority, and women’s inferiority and expected submissiveness. Women’s acceptance of traditional gender norms within the relationship promoted self-blame, self-sacrifice and acceptance of the situation, including tolerating his gambling and the associated abuse. The women tended to assume responsibility for trying to fix problems in the relationship, including those caused by gambling [45]. Some blamed themselves and made more sacrifices, which may also reflect the negative self-perception often caused by abuse [48].

This gender hierarchy within the relationship was enforced by male partners, who were typically described as misogynistic, controlling, entitled and selfish, reflecting strong gendered drivers of violence against women [39, 40, 49, 50]. Male partners assumed a perceived entitlement to control decision-making, subordinate their partner, and use violence against her. Gambling became another relationship stressor that exacerbated violence. Perpetrators often weaponised their female partner’s gambling to justify their violence. Where the man had a gambling problem, the behavioural drivers of problem gambling [51] interacted with, and intensified, his controlling, coercive and abusive behaviours. This resulted in violence linked to his preoccupation with gambling, frustration when unable to gamble, anger over gambling losses and desperation to acquire money. All partners with a gambling problem reportedly financially abused the woman. Problem gambling provides a powerful incentive for financial abuse, so these two issues commonly co-occur and may be enforced by psychological and physical violence [23, 52, 53]. Participants described violent responses when they failed to meet their partner’s needs, challenged his authority, questioned his gambling, could not provide money for gambling, or sought to pursue their own interests.

Expectations to keep domestic problems private meant that most women kept the abuse and the gambling problem hidden due to shame, and an expectation that family and friends would not be helpful, reflecting cohort effects found in previous research [30, 44, 54–59]. While lacking support, these women often struggled with the competing choices of staying for the sake of their children and to retain the couple’s shared history and financial security, and their own ongoing safety and wellbeing [35, 48, 57]. Some women stayed and accepted the compromises this entailed [54, 55], reflecting that the complexities surrounding IPV in long-term relationships are amplified by financial dependence, strong emotional ties, and sentimental attachments [56, 57]. Some legal determinants of health also deterred the women from leaving, including fear of losing custody of the children, or that their partner’s abusive behaviour and gambling addiction would undermine their children’s welfare if he gained access after separation. The financial stresses from gambling meant that many women had no realistic capacity to leave the relationship, especially if they had dependent children, and there was no legal recourse for financial abuse within marriage. Most women who eventually left the relationship stayed longer than they wished, extending their experiences of violence. Services supporting older women need to be sensitive to the strong gendered norms, financial stress and pressures felt by this cohort; respond in ways that are empowering but enable them to stay physically and emotionally safe; and find creative ways to assist women’s safety when they choose to stay in an abusive relationship. These women’s experiences also emphasise the critical importance of continued efforts to reduce gender inequality, since hierarchical relationships with rigid gender expectations increase the risk of IPV against women [1, 3].

Societal-level influences at the time also promoted a traditional view of marriage as a permanent relationship with highly gendered roles [45, 56]. These values were reinforced during these women’s upbringing [30, 44–[45, 54, 58]–59]. Many women felt pressured to stay married, silently accept their “lot”, not question their husband’s authority and abuse, and maintain the façade of a perfect family [44, 45]. Tolerating and concealing domestic problems also included hiding the gambling problem and its impacts. This silence surrounding IPV and gambling was also reflected in the lack of public discourse about these issues. Consequently, many women did not recognise their partner’s behaviour as IPV, including the abuse related to gambling, and accepted the prevailing view that husbands had a right to control their wives. Some women with a gambling problem felt they deserved the abuse. Many women thought IPV included only severe physical abuse, so they might see themselves as “merely” being mistreated [56, 57]. They downplayed emotional, verbal and financial abuse, which was often linked to his gambling, and did not consider seeking help. There was also no public discourse about problem gambling at the time and that it can exacerbate controlling, coercive and abusive behaviours [6]. Instead, the women, their families and friends, services and institutions saw gambling as a normal, harmless pastime. When women did seek social support, family and friends might dismiss their experiences, maintain a shared silence about the perpetrator’s abuse and his gambling, or pressure the woman to persevere [56]. These issues point to the importance of tailored community awareness about IPV and problem gambling, how to help affected women, and professional sources of help.

In terms of period effects, the systems responses at that time were poor, reflecting little professionalisation in addressing IPV and no consideration of the role of gambling in exacerbating abuse. There were no DFV services, financial support or childcare to help women escape violent relationships and no services for problem gambling. Police, justice, healthcare and other services had limited understanding of IPV and gambling, and their responses were often inadequate [36, 37, 44]. Police responses were based on a violent incident model that views IPV in terms of discrete assaults and serious injury, while ignoring patterns of coercive control that are more common, enduring and damaging [60–63]. Some women reported that police stereotyped them as nuisance “repeaters”, not recognising their abuse was ongoing [62]. As still found today [64], DVOs were often ineffective and, if breached, women were sometimes blamed and deterred from laying charges. These experiences reflect the lack of IPV legislation at that time, limitations to police responses in law, and a failure to take violence against women seriously and to protect their safety. When women did seek help, many encountered gendered responses, reflecting the accepted practices at that time. Some healthcare professionals blamed the women, trivialised the abuse and the gambling, or did not help them recognise the abuse or the gambling [65]. As found previously [35, 54], police sometimes privileged the perpetrator’s account over the woman’s, deterred her from pressing charges, treated her as an hysterical annoyance, or did not take the violence seriously. Negative experiences of help-seeking can deter future help-seeking, even though services may have improved [32, 34–36]. The women’s experiences demonstrate how inadequate support services, lack of legal recourse, and unsupportive and gendered institutional responses can compound the effects of gambling-related IPV and further increase risks to women’s safety.

### Limitations

The non-probability sample was not necessarily representative of women affected by gambling-related IPV. We prioritised gaining rich detailed insights from a small group of women who could safely and willingly share their experiences, over gaining a large representative sample. For ethical and safety reasons, we only interviewed women who had received professional support for IPV or gambling; different insights may be gained from women who have not sought professional help. We also only interviewed women who had come to acknowledge their experiences as IPV; women who have experienced IPV but have not recognised or named it as such may provide different perspectives. While the sample contained women who varied by cultural group, socio-economic status, health status and location, the small sample size precluded analyses based on these diverse characteristics. Future research would benefit from exploring women’s past experiences from these more nuanced perspectives. Few participants were experiencing IPV in their older age, as most had left the abusive relationship many years earlier. Research with older women with recent experience of IPV is needed to explore age effects on IPV [30].

## Conclusions

From a prevention perspective, the study’s findings point to the critical importance of reducing gender inequality to reduce male partner violence towards women, including violence linked to gambling. This requires longer-term attitudinal, systemic, structural and normative change at multiple levels of the social ecology. However, women being impacted by gambling-related IPV, including the legacy of past abuse, also need services and systems that effectively cater to their needs, recognise the many forms and patterns of abuse involved, understand the historical and contextual factors that underlie and exacerbate IPV, and recognise the role of gambling in intensifying violence. Supporting women impacted by gambling-related IPV presents distinctive challenges and requires a non-judgmental, tailored approach that recognises the intersecting issues in each woman’s situation.

The widespread availability and promotion of gambling and the ongoing failure to reduce gambling problems in Australia [66–68] continue to amplify the abuse of women and their children. Practical support to manage their finances and build self-efficacy can help women affected by gambling-related IPV to contain the harm [69, 70]. More critically however, policy, regulatory and gambling industry changes are essential to reduce problem gambling and its exacerbation of violence against women.

## Data Availability

The datasets generated and analysed during the current study are not publicly available because the participants consented for their data to be used only by the research team. Please contact the corresponding author to request access to the data.

## List of abbreviations

DV: Domestic violence
DVO: Domestic violence order
IPV: Intimate partner violence
WMG: woman, man’s gambling
WFA: woman, financial abuse
WWG: woman, woman’s gambling
